# Comparative Distribution Projections of Hydrophilic and Xerophytic Invasive Species in Turkey Under CMIP6 Climate Scenarios

**DOI:** 10.1002/ece3.74172

**Published:** 2026-08-15

**Authors:** Nihat Tursun, İlhan Üremiş, Soner Soylu, Ahmet Uludağ

**Affiliations:** ^1^ Department of Plant Protection, Faculty of Agriculture Malatya Turgut Özal University Battalgazi Malatya Türkiye; ^2^ Department of Plant Protection, Faculty of Agriculture Hatay Mustafa Kemal University Antakya Hatay Türkiye; ^3^ Department of Plant Protection, Faculty of Agriculture Çanakkale Onsekiz Mart University Çanakkale Türkiye

**Keywords:** biological invasion, climate change, CMIP6, *Eichhornia crassipes*, MaxEnt, *Solanum elaeagnifolium*

## Abstract

Climate change imposes varying pressures on the potential distribution of invasive alien plant species, depending on their specific ecological strategies and physiological tolerances. In this study, the projected potential ranges within Turkey of the hydrophilic *Eichhornia crassipes* (water hyacinth) and the markedly xerophytic *Solanum elaeagnifolium* (silverleaf nightshade) were compared under several CMIP6 climate scenarios: SSP2‐4.5, SSP3‐7.0 and SSP5‐8.5. Species distribution models were produced using an ensemble modelling framework combining Maximum Entropy (MaxEnt), Random Forest (RF) and Boosted Regression Trees (BRT) in the R statistical computing environment, yielding excellent predictive performance for both taxa. Model outputs revealed strongly divergent, indeed opposing, spatial responses between the two species. Rather than persisting stably within its current range, *E. crassipes* is projected to undergo a severe contraction of its suitable habitat across all emission scenarios, as increasing drought severity and the degradation of wetland habitats progressively outweigh any potential benefit from milder winters, restricting the species to highly fragmented coastal micro‐refugia. In contrast, *S. elaeagnifolium* displays a more complex, non‐linear trajectory: an initial, pronounced expansion into the interior agricultural basins of the Aegean hinterland and Southeastern Anatolia through the mid‐century, followed by a marked contraction by 2100 as extreme thermal and arid conditions exceed the species' physiological tolerance limits, particularly under the higher emission scenarios. These results demonstrate that climate change does not confer a uniform range‐expansion advantage on invasive taxa. Instead, effective management requires dynamic, species‐specific strategies—including early warning systems, strict quarantine measures, and spatially explicit, climate‐informed risk mapping that account for both the timing and directionality of range shifts.

## Graphical abstract

Using an ensemble species distribution modelling framework (MaxEnt, Random Forest and Boosted Regression Trees), we compared the projected climate‐driven range shifts of two ecologically contrasting invasive plants in Turkey—the hydrophilic *Eichhornia crassipes* and the xerophytic *Solanum elaeagnifolium* —under three CMIP6 scenarios (SSP2‐4.5, SSP3‐7.0, SSP5‐8.5). Results reveal opposing trajectories: *E. crassipes* is projected to undergo severe habitat contraction across all scenarios due to worsening hydrological stress, whereas *S. elaeagnifolium* shows a pronounced mid‐century expansion into interior agricultural regions followed by a marked contraction by 2100 as extreme aridity exceeds its physiological tolerance. These findings show that climate change does not uniformly favour invasive species expansion and highlight the need for species‐specific, time‐sensitive management strategies.

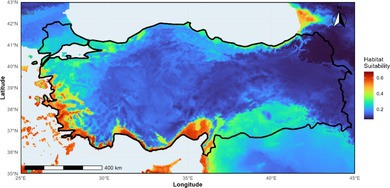

## 1 Introduction

The drivers of biodiversity loss and the associated degradation of ecosystem services should no longer be viewed as separate, isolated processes, but as mutually reinforcing, amplifying global crises. Global climate change and invasive alien species (IAS) are two of the primary proximate drivers of biodiversity decline; furthermore, their synergistic interaction exerts a compounded, dual pressure on ecosystem integrity (Bellard et al. 2013; IPBES 2019). Climate change—evidenced by rising ambient temperatures, altered precipitation patterns and increased frequency and intensity of extreme weather events—pushes native species towards or beyond their physiological tolerance limits, reducing their competitive fitness. This ecological imbalance facilitates the creation of suitable niche space for invasive taxa with broad ecological ranges and marked phenotypic plasticity (Walther et al. 2002; Hellmann et al. 2008). Conversely, established invasive species undermine ecosystem resilience, making biotic communities more vulnerable to climatic disturbances and creating a harmful feedback loop between climate change and biological invasion (Diez et al. 2012). The synergy between these factors significantly hinders the conservation of native biodiversity and requires the adoption of multidimensional, integrative approaches in contemporary ecosystem management.

The interaction between climate change and biological invasions is particularly pronounced across climatic transition zones, especially within the Mediterranean Basin. The Mediterranean Region is characterised by heightened vulnerability to climate forcing, with a rate of warming that exceeds the global average and an increased risk of severe, prolonged drought (Cramer et al. 2018; IPCC 2021). Turkey, due to its pivotal position at the European–Asian biogeographic transition zone, its location at the intersection of the Mediterranean, Iran–Turan and Euro–Siberian phytogeographic regions and the extensive area of its agricultural production landscapes, is consistently ranked among nations facing elevated risk from climate‐mediated biological invasions (Giorgi and Lionello 2008; Bellard et al. 2013; Walther et al. 2002; IPCC 2021).

The impact of climate change on invasive species is complex. Depending on their physiological, morphological and ecological characteristics, their geographical range may expand, contract or shift. Invasive species with a xerophytic strategy, such as *Solanum elaeagnifolium* Cav. (silver leaf nightshade), are predisposed to exploit rising temperatures and increased aridity, thereby gaining a competitive advantage and facilitating range expansion. Conversely, aquatic or hygrophilous taxa such as *Eichhornia crassipes* (Mart.) Solms may experience regional retractions in response to local hydrological deficits or supra‐optimal thermal stress (Petitpierre et al. 2012; Early and Sax 2014). Within this context, comparative modelling of spatially explicit responses to climate forcing by species with contrasting ecological strategies is a critical prerequisite for effective horizon scanning—initiatives aimed at detecting emerging biological threats while they remain manageable. Moreover, high‐resolution climatic projections provide essential baseline data for integration into international IUCN protocols, including the Environmental Impact Classification for Alien Taxa (EICAT) and the Socio‐Economic Impact Classification for Alien Taxa (SEICAT), both of which standardise assessments of the detrimental consequences invasive species impose on ecosystems and human well‐being. Spatially explicit forecasting of future range dynamics thus transforms risk assessment from a reactive approach into a proactive management framework, equipping policymakers with a robust decision‐support tool.

*Solanum elaeagnifolium* , a focal taxon of this study, is a species native to the Americas that is now recognised as a highly invasive and exceptionally problematic weed, noted for its pronounced adaptive capacity and competitive vigour. It is undergoing rapid range expansion across a broad geographic area, particularly in Europe and the Mediterranean Basin. The species has been designated for quarantine listing by the European and Mediterranean Plant Protection Organization (EPPO) and is consistently ranked among high‐risk invasive alien plants, largely due to the substantial economic losses it causes in agricultural production systems (EPPO 2007). Its deep, extensively branched vegetative root system, formidable regenerative capacity and marked drought tolerance underpin *S. elaeagnifolium* 's ability to compete vigorously with major agricultural crops, including cash crops like cotton and staple crops like maize and wheat, for water and essential soil nutrients. These attributes highlight the species' pronounced adaptation to water‐deficit stress, a capacity conferred by its expansive root architecture (Roberts and Florentine 2022; Formozis et al. 2021).

The current distribution of *S. elaeagnifolium* in Turkey is largely restricted to the Mediterranean and Aegean coastal regions (Uludağ et al. 2024). With rising temperatures and increasing aridity, it is expected that the ecological tolerance of this species may expand significantly, enabling its spread into interior and semi‐arid areas. This development suggests not only a geographical extension of its potential range but also an increased risk of greater impacts on previously unaffected agroecological systems. In contrast, *E. crassipes* (water hyacinth) is globally recognised as one of the most damaging aquatic invaders, with an exceptional ability to cause severe disruption in freshwater ecosystems. Due to its high rates of both generative and vegetative reproduction, this species can quickly form extensive, dense surface mats that greatly reduce light penetration in the water column, suppressing photosynthesis and causing critical declines in dissolved oxygen levels. These processes compromise the structural and functional integrity of aquatic habitats, leading to reduced biological diversity and posing serious threats to the ecological sustainability of water resources (Villamagna and Murphy 2010; Uremis et al. 2014). Additionally, the dense, high‐biomass canopy formed at the water surface significantly increases evaporative losses, thereby exacerbating water shortages; at the same time, it physically blocks irrigation channels and water conveyance infrastructure, restricting hydraulic flow and severely impairing the operational efficiency of these systems (Téllez et al. 2008). Owing to its tropical origin, the species is highly sensitive to low temperatures, with frost events serving as a particularly important limiting factor. Its distribution is therefore closely linked to the year‐round presence of standing water and suitable thermal conditions (Hilooğlu and Sözen 2017). It is still unclear how increasing drought trends and the quantitative decline in water resources, as projected by climate change scenarios, will affect the future potential distribution of this hydrophilic species.

Most studies have primarily assessed climate‐induced range dynamics of invasive alien species by examining individual taxa in isolation. However, comparative studies that clarify how invasive species with markedly different ecological requirements—such as xerophytic and hydrophilic strategies—display divergent spatial responses within the same geographic area and under identical climate projections are notably scarce. In this context, comparative analyses using species distribution modelling frameworks offer significant potential to enhance our understanding of the complex impacts of climate change on biological invasions (Elith and Leathwick 2009). Therefore, investigating the responses of invasive taxa to climatic changes not only through single‐species assessments but also by comparing species with contrasting ecological strategies is crucial. The existing literature has mainly focused on taxa with broadly similar physiological traits; studies that explicitly contrast the spatial responses of water‐dependent and drought‐adapted invaders under a shared set of climate scenarios are particularly limited, especially within the Mediterranean Basin.

The main objective of this study is to apply an ensemble modelling framework (incorporating Maximum Entropy, Random Forest and Boosted Regression Trees) alongside contemporary climate projections from the Coupled Model Intercomparison Project Phase 6 (CMIP6)—specifically the SSP2‐4.5, SSP3‐7.0 and SSP5‐8.5 scenarios—to achieve three integrated aims: (i) delineate the current potential distribution of the hydrophilic *E. crassipes* and the xerophytic *S. elaeagnifolium* across Turkey; (ii) project shifts in habitat suitability for these species at three temporal horizons (2050, 2070 and 2100); and (iii) evaluate the spatially explicit, divergent responses of these two invasive taxa, which represent fundamentally contrasting ecological strategies, to anticipated climate forcing.

Moving beyond conventional single‐species assessments, this research directly compares the distinct range‐shift trajectories of a hydrophytic and a xerophytic invader subjected to identical climate forcing. The underlying hypothesis is that global warming does not confer a uniform expansion advantage across all invasive alien flora. Instead, species‐specific physiological thresholds and habitat connectivity requirements are expected to generate discrete spatial outcomes, conceptually characterised as having either ‘limited additional climatic expansion potential’ or ‘directional spread’. Consequently, this study advocates a more nuanced risk assessment framework that extends beyond quantitative changes in suitable area. It incorporates the directionality of range movement, the intrinsic dynamics of spatial shift and the functional attributes of recipient ecosystems. By adopting this integrated perspective—simultaneously analysing spatial directionality and functional risk patterns within a unified analytical framework—this investigation constitutes one of the pioneering comparative assessments at the national scale for Turkey, explicitly linking hydrophilic and xerophytic invasion dynamics.

## 2 Material and Methods

### 2.1 Study Area and Occurrence Data

This study covers the whole of Turkey, a territory that serves as a crucial biogeographic bridge between Asia and Europe and lies at the intersection of the Euro‐Siberian, Mediterranean and Iran‐Turan phytogeographic regions (Şekercioğlu et al. 2011). The country's significant biogeographic heterogeneity is further influenced by the dominant climatic dynamics of the Mediterranean Basin, a region widely recognised as one of the main ‘hotspots’ expected to experience the most marked and rapid effects of global climate change (Giorgi and Lionello 2008; Cramer et al. 2018). This unique geographic and bioclimatic position makes Turkey an ideal model system for evaluating climate‐driven range dynamics and for assessing the cascading ecological impacts of biological invasions on native ecosystems.

The focal taxa for this investigation are *E. crassipes* , a species causing severe environmental and economic disruptions in aquatic ecosystems, and *S. elaeagnifolium* , which demonstrates strong invasive potential in arid and semi‐arid agricultural landscapes. These two species have fundamentally different ecological requirements and contrasting invasion strategies, making them suitable model organisms for a comparative analysis of their responses to climatic pressures.

Global occurrence records for the target species were obtained from the Global Biodiversity Information Facility (GBIF) database (GBIF.org 2026) and supplemented by a comprehensive review of recent peer‐reviewed literature. The raw data were subjected to strict quality control protocols, including verification of coordinate accuracy, assessment of taxonomic consistency and identification and removal of duplicate entries. To reduce sampling bias and spatial autocorrelation, the occurrence records were spatially rarefied using a grid of 10 km × 10 km cells, retaining only one unique record per grid cell (Boria et al. 2014; Elith et al. 2011). After this spatial filtering, the final datasets used for analysis comprised 6245 spatially independent and validated occurrences for *E. crassipes* and 3812 for *S. elaeagnifolium* .

### 2.2 Bioclimatic Data and Scenarios

To model the contemporary potential distributions of the focal taxa, 19 bioclimatic variables were obtained from the WorldClim version 2.1 repository (Fick and Hijmans 2017). These variables represent long‐term climatological normals for both temperature and precipitation regimes and are widely used in delineating species' ecological niches. The climatic data used in this study were acquired at a high spatial resolution of 5 arc‐minutes (~10 km at the equator) to capture fine‐scale ecological variability, directly addressing the complex topography of the Mediterranean Basin. To avoid multicollinearity and subsequent model overfitting, a Pearson correlation analysis and variance inflation factor (VIF) assessment were conducted on the initial 19 bioclimatic variables. Only variables with a Pearson correlation coefficient of |*r*| < 0.75 and VIF < 5 were retained for the final ensemble modelling process (Figure S1). Future climate projections were sourced from datasets produced under the Coupled Model Intercomparison Project Phase 6 (CMIP6), developed within the framework of the IPCC Sixth Assessment Report (AR6) (Eyring et al. 2016; IPCC 2021). Among the available general circulation models (GCMs), the high‐resolution MPI‐ESM1‐2‐HR (Max Planck Institute Earth System Model) was selected. This model has demonstrated robust and accurate simulation capabilities for temperature and precipitation extremes, particularly within semi‐arid climatic zones such as the Mediterranean Basin, and has shown strong performance in recent species distribution modelling (SDM) applications (Müller et al. 2018; Zabihi and Ahmadi 2024).

To assess the potential impacts of climate forcing across a range of plausible future trajectories, three distinct shared socioeconomic pathways (SSPs) were selected for analysis:

- SSP2‐4.5: A ‘middle‐of‐the‐road’ scenario, based on intermediate levels of radiative forcing and representing a trajectory in which current socioeconomic and technological trends continue without significant change.
- SSP3‐7.0: A high‐emission scenario marked by renewed regional rivalries, fragmented governance and the implementation of only limited or restricted climate policy measures.
- SSP5‐8.5: A fossil fuel‐intensive development pathway, representing the most severe climate change scenario considered here, in which anthropogenic emissions continue to rise unchecked throughout the 21st century.

The key indicators for projected global mean temperature increases and atmospheric CO_2_ concentrations through 2100 for the SSP scenarios used in this study are summarised in Table 1.

**TABLE 1:** Projected global mean temperature increases and atmospheric CO_2_ concentrations through the year 2100 under the SSP scenarios used within the framework of IPCC AR6.

Scenario	Description	Projected global mean temperature increase by 2100 (relative to pre‐industrial levels)	Atmospheric CO_2_ by 2100 (ppm)
SSP2‐4.5	Middle of the road (moderate emissions)	~2.7°C	~600
SSP3‐7.0	Regional rivalry (high emissions)	~3.6°C–3.7°C	~850
SSP5‐8.5	Fossil‐fuelled development (very high emissions)	~4.4°C	~1135

*Note:* Future projections were generated for three distinct temporal windows representing the near‐term (2041–2060), mid‐term (2061–2080) and long‐term (2081–2100) impacts of climate change. The resulting climatic layers were then used as input variables for the species distribution models.

### 2.3 Species Distribution Modelling

The potential habitat suitability of the focal taxa was estimated using an ensemble modelling framework combining three diverse machine‐learning algorithms: Maximum Entropy (MaxEnt), Random Forest (RF) and Boosted Regression Trees (BRT). This multi‐model consensus approach significantly reduces algorithm‐specific biases and uncertainties associated with single‐model projections (Araújo and New 2007). Individual algorithms, such as MaxEnt which is specifically designed for presence‐only data (Phillips et al. 2006), were configured to capture the complex species–environment relationships from presence‐only data. For RF, 1000 classification trees were grown per run (ntree = 1000) using the default number of predictors sampled at each split; for BRT, models were fitted with 1000 trees, an interaction depth of 3 and a shrinkage (learning rate) of 0.01. The continuous suitability surfaces produced by the three calibrated algorithms were then combined into a single consensus projection by calculating, for each grid cell, the unweighted arithmetic mean of the three standardised (0–1) outputs. This ensemble‐mean surface, rather than any single algorithm's output, was used for all subsequent thresholding, mapping and range‐shift analyses.

All spatial analyses and modelling procedures were conducted within the R Statistical Computing Environment (v4.3.2) (R Core Team 2023). The modelling workflow was implemented using the following R packages:

- dismo (v1.3‐14): For the execution of the MaxEnt algorithm and associated model calibration procedures (Hijmans et al. 2017);
- terra (v1.7‐55): For the manipulation of gridded climatic data in raster format, coordinate reference system transformations and ancillary spatial analyses (Hijmans 2023);
- rJava: To provide the necessary Java integration enabling the seamless operation of the MaxEnt software executable within the R environment;
•randomForest & gbm: For the execution of the Random Forest and Boosted Regression Trees algorithms, respectively;
- ggplot2, tidyterra & ggspatial: Specifically employed for advanced cartographic visualisation, including the generation of high‐resolution suitability maps incorporating rigorous spatial standards (e.g., scale bars, orientation arrows and georeferenced grids) as required for robust spatial ecology reporting.

To ensure robust calibration and prevent model overfitting, MaxEnt algorithms were configured using linear, quadratic and hinge feature classes (LQH) with a regularisation multiplier set to 1.0. Furthermore, to assess model stability and predictive consistency, 10 replicate runs were performed for each species.

Model performance was evaluated using a randomly selected 25% subset of the occurrence data reserved for testing. Model accuracy was quantified using both the area under the receiver operating characteristic curve (AUC) and the true skill statistic (TSS). Although AUC evaluates the discriminatory capacity of the models, TSS was explicitly included to account for prevalence‐independent predictive accuracy, providing a more robust and comprehensive validation framework (Allouche et al. 2006). Models with AUC values above 0.90 were classified as ‘excellent’, those in the 0.70–0.90 range as ‘good’ and values below 0.70 as indicative of ‘poor’ predictive performance (Swets 1988). It should be noted that the spatial rarefaction of occurrence records (Section 2.1) reduces the influence of spatially clustered sampling on model calibration, but it does not by itself constitute a spatially structured cross‐validation of predictive performance: the random 25% test partition described above may still contain test points that are spatially proximate to, and therefore not fully independent of, training points, potentially inflating the reported AUC/TSS values to some degree. A fully spatially blocked cross‐validation (e.g., checkerboard or k‐means spatial blocking) was not implemented in the present study and is identified as a priority methodological refinement for future work; the AUC/TSS values reported here should accordingly be interpreted as an upper bound on true predictive transferability rather than as a spatially independent validation.

The baseline distribution maps generated for the contemporary period (1970–2000) represent not the actual extant occurrences of the species, but the maximum potential climatic suitability—that is, the geographic envelope where bioclimatic conditions are currently conducive to their establishment. To evaluate invasion risk across the broadest possible spatial framework and facilitate a clear comparative assessment of climate change impacts, threshold suitability values of 0.35 for *S. elaeagnifolium* and 0.30 for *E. crassipes* were adopted. This analytical approach enabled the projection of invasion risk to include ‘early warning’ zones, areas that are ecologically and climatically suitable but not necessarily currently occupied by the species, thus providing a more precautionary and forward‐looking assessment.

### 2.4 Spatial Change Analysis

To quantitatively assess future invasion risk, the continuous habitat suitability surfaces (ranging from 0 to 1) generated by the ensemble models were converted into binary (presence/absence) distribution maps using species‐specific threshold values. Threshold selection was guided by the ‘maximum sum of sensitivity and specificity’ (MaxSS) approach, a methodology widely recognised for maximising predictive accuracy while minimising error rates in species distribution modelling frameworks (Liu et al. 2005; Jiménez‐Valverde and Lobo 2007). Following this principle and considering the differing ecological tolerances of the focal taxa and the predictive balance of the respective models, a threshold value of 0.30 was applied for *E. crassipes* , whereas a value of 0.35 was used for *S. elaeagnifolium* —a species with a demonstrably broader ecological amplitude. This interspecific threshold differentiation aligns with the use of species‐specific thresholding strategies that reflect the fundamental structural differences in habitat requirements between aquatic and xerophytic taxa (Nenzén and Araújo 2011).

Spatial change ratios were calculated on a pixel‐by‐pixel basis using map algebra, in which the binary (0 = unsuitable, 1 = suitable) baseline projection map representing current conditions was subtracted from each corresponding binary projection map generated under the suite of future climate scenarios (Thuiller et al. 2005). In the resulting difference maps, positive values (+1) indicate newly acquired potential habitats, representing emergent areas of expansion risk due to climate change, whereas negative values (−1) denote regions where habitat suitability is projected to be lost (range contraction). A value of zero (0) marks areas of relative stability, where the species' potential presence is expected to persist or remain unchanged (Peterson et al. 2011). This quantitative framework enables a clear and directly comparable interpretation of climate‐driven spatial risk patterns and the underlying dynamics of range shift.

### 2.5 Risk Assessment Framework

Risk assessment was conducted not solely by quantifying changes in the extent of potential distributional areas but through an integrated evaluative framework that incorporates spatial contraction and expansion, geographic displacement and habitat fragmentation as interrelated components. The baseline distributions of the focal taxa, representing their potential ranges under current climatic conditions, were systematically compared with those projected under the ensemble of future climate scenarios. Risk patterns were delineated based on relative shifts in habitat suitability.

Risk categories were defined according to both the direction and spatial configuration of change within suitable areas. Regions showing pronounced suitability loss relative to current potential distributions were classified as high risk. Areas characterised by a poleward or altitudinal displacement of suitable habitat—where the climatic envelope shifts, leaving previously occupied zones increasingly marginal—were designated as moderate risk. Conversely, regions where existing suitable areas are projected to be largely retained were assigned to the low‐risk category. This approach enables a spatially holistic and ecologically nuanced interpretation of climate‐mediated biological risks (Araújo et al. 2011).

### 2.6 Mapping and Visualisation

Model outputs were processed in a Geographic Information Systems (GIS) environment and converted into potential distribution maps covering all of Turkey. To adhere to rigorous cartographic standards and ensure spatial precision, all final maps were augmented with geographic coordinate grids (latitude and longitude), scale bars and north arrows. The continuous habitat suitability surfaces were reclassified into discrete categories of suitable and unsuitable areas using species‐specific threshold criteria. Baseline and future projection maps were standardised with the same classification scheme and uniform colour ramp, enabling direct visual comparison across scenarios and time periods. This standardisation ensures that the spatial changes and associated risk patterns for each taxon under different climate forcing scenarios remain visually comparable and analytically manageable.

## 3 Results

The species distribution models constructed using the ensemble modelling framework (incorporating MaxEnt, Random Forest and Boosted Regression Trees) demonstrated high statistical fidelity for both invasive taxa. As detailed in Table 2, all individual algorithms and the final consensus models exhibited excellent predictive performance, supported by robust Area Under the Curve (AUC) and True Skill Statistic (TSS) metrics. The spatial projections generated after model validation showed that the hydrophilic *E. crassipes* and the xerophytic *S. elaeagnifolium* are likely to pursue fundamentally divergent, indeed opposing, range expansion strategies in response to Turkey's projected future climate trajectories under the SSP2‐4.5, SSP3‐7.0 and SSP5‐8.5 scenarios. The findings are presented below in a comparative framework, with consideration given to the severity gradients among scenarios and the distinct temporal windows examined.

**TABLE 2:** Predictive performance and validation metrics of individual algorithms and the final ensemble model for the target invasive alien species.

Species	Model algorithm	AUC	TSS
*Eichhornia crassipes*	MaxEnt	0.93	0.78
	Boosted Regression Trees (BRT)	0.94	0.81
	Random Forest (RF)	0.97	0.86
	Ensemble Model	0.96	0.84
*Solanum elaeagnifolium*	MaxEnt	0.98	0.91
	Boosted Regression Trees (BRT)	0.99	0.93
	Random Forest (RF)	0.99	0.94
	Ensemble Model	0.99	0.94

For *Eichhornia crassipes* , the Random Forest algorithm yielded the highest discriminative capacity with an AUC of 0.97, followed by BRT (0.94) and MaxEnt (0.93). The integrative ensemble model for *E. crassipes* maintained an excellent accuracy standard with an AUC of 0.96 and a TSS of 0.84. For *Solanum elaeagnifolium* , all modelled algorithms exhibited near‐perfect predictive metrics. Both Random Forest and Boosted Regression Trees achieved a top‐tier AUC value of 0.99, whereas the individual MaxEnt model scored 0.98. The validated TSS scores for *S. elaeagnifolium* consistently exceeded the 0.90 threshold, peaking at 0.94 within the final consensus ensemble framework, thereby validating the exceptional transferability and scientific rigour of the projections.

### 3.1 Overview of Spatial Responses of the Focal Taxa

The general spatial distribution patterns of *E. crassipes* and *S. elaeagnifolium* across the climate scenarios and time horizons examined are summarised in Table 3. This summary offers a comparative overview of the observed tendencies, including range stability, expansion and directional displacement, providing a framework for the detailed spatial change analyses discussed in the following subsections.

**TABLE 3:** Summary of spatial distribution shifts observed for *Eichhornia crassipes* and *Solanum elaeagnifolium* under different climate scenarios and across distinct time periods.

Species	Scenario	2050	2070	2100	Overall spatial response
*E. crassipes*	SSP2‐4.5	Decrease	Stable	Stable	Coastal compression
*E. crassipes*	SSP3‐7.0	Decrease	Stable	Stable	Limited expansion potential
*E. crassipes*	SSP5‐8.5	Decrease	Stable	Stable	Hydrological constraint
*S. elaeagnifolium*	SSP2‐4.5	Slight expansion	Moderate expansion	Expansion	Inland displacement
*S. elaeagnifolium*	SSP3‐7.0	Expansion	Pronounced expansion	Pronounced expansion	Invasion front
*S. elaeagnifolium*	SSP5‐8.5	Pronounced expansion	Very pronounced expansion	Maximal expansion	Drought advantage

#### 3.1.1 *Eichhornia crassipes*

##### 3.1.1.1 Current Distribution

**FIGURE 1:**
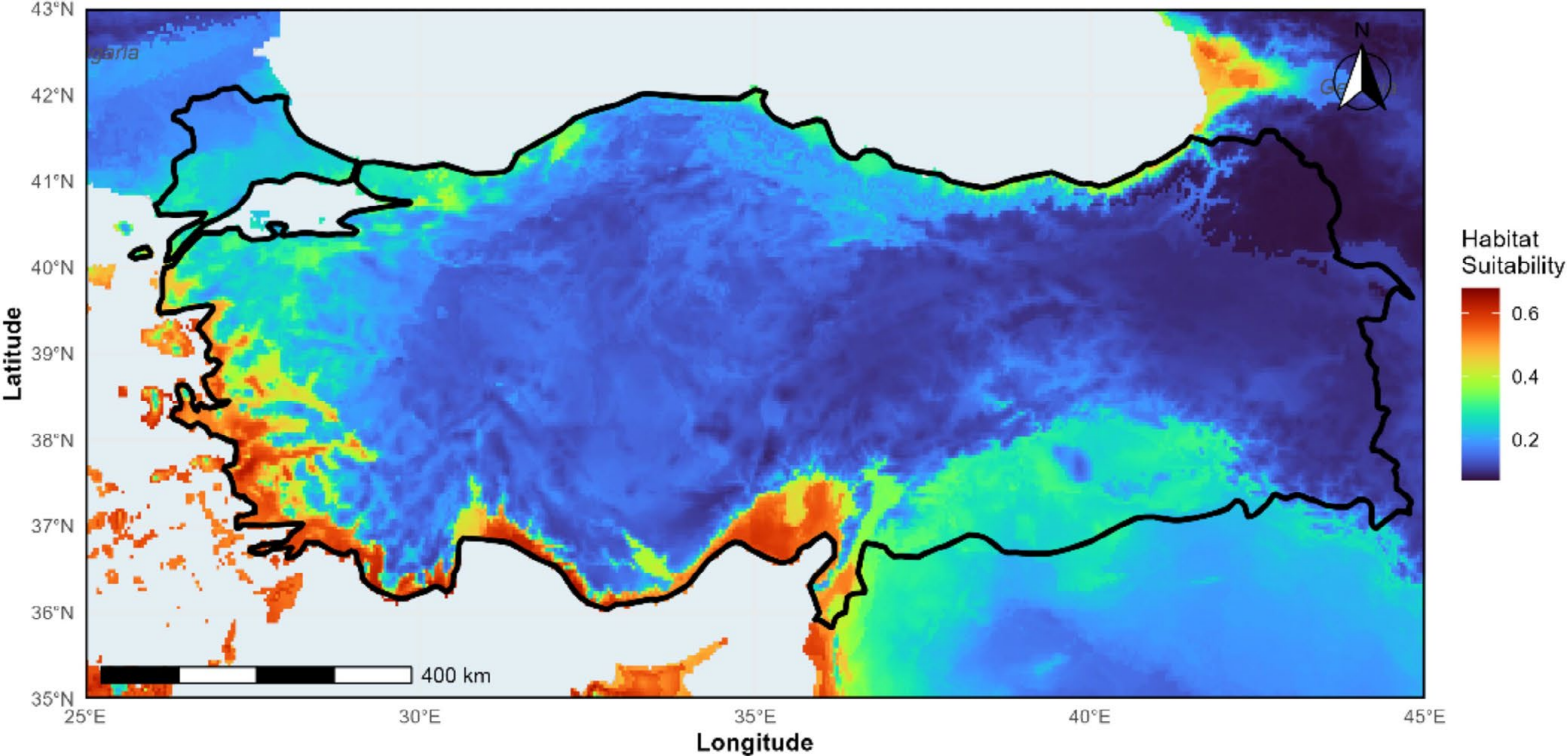
Current potential habitat suitability for *Eichhornia crassipes* in Turkey under baseline climatic conditions. The colour gradient from dark blue to red represents increasing habitat suitability (ranging from 0 to 1.0), with red areas indicating optimal climatic conditions for establishment.

**FIGURE 2:**
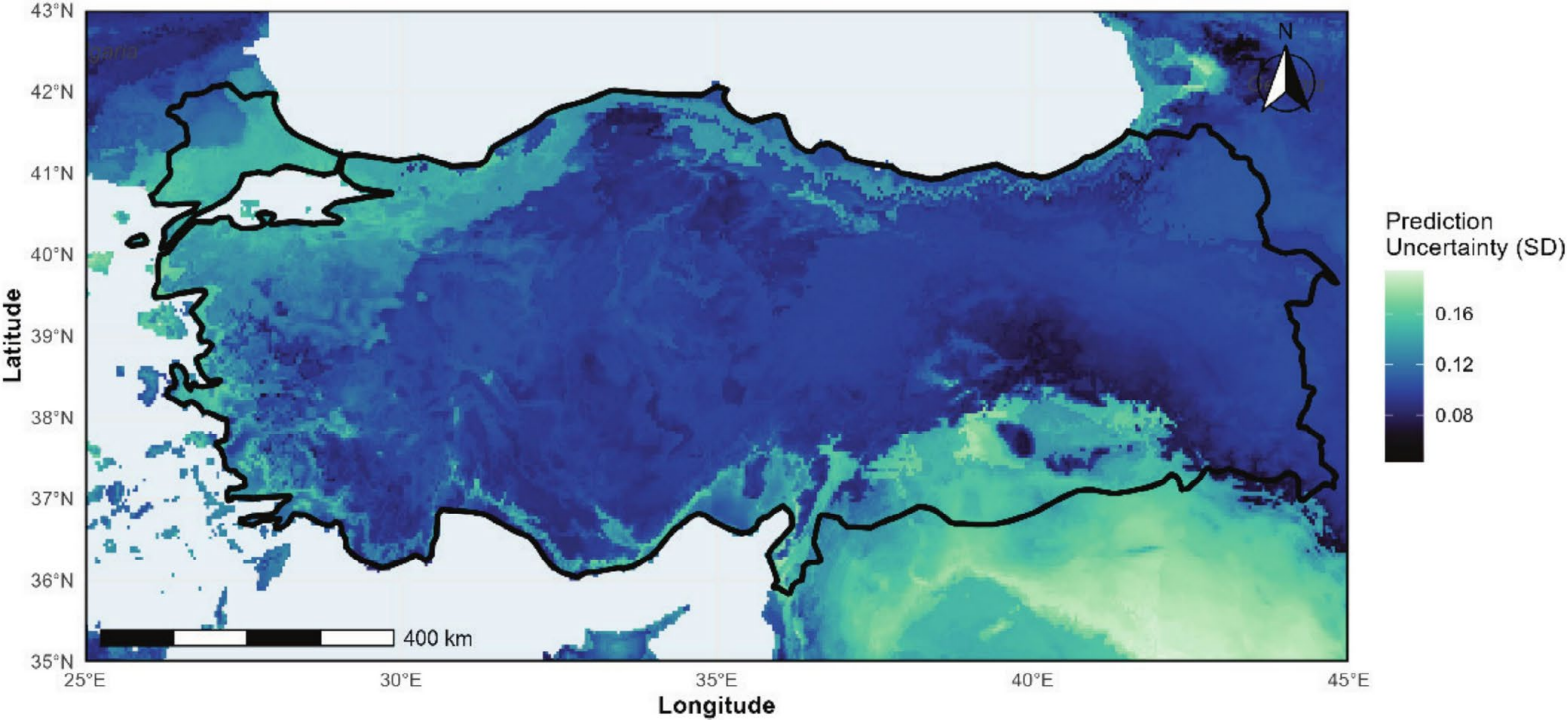
Spatial prediction uncertainty (standard deviation) associated with the ensemble modelling framework for *Eichhornia crassipes* . Higher standard deviation values (lighter colours) indicate geographic regions with greater variance and lower consensus among the constituent machine‐learning algorithms (MaxEnt, RF and BRT) whereas darker areas indicate high model agreement.

Initial model outputs (Figure 1) indicate that, under baseline climatic conditions, habitat suitability for *Eichhornia crassipes* reaches high to very high values (0.8–1.0) along the Aegean (İzmir, Muğla) and Mediterranean (Antalya, Mersin, Adana) coastal littorals. The robustness of these baseline predictions is further validated by the spatial uncertainty analysis (Figure 2), which demonstrates high inter‐model consensus (low standard deviation) across these core suitable coastal zones. However, the topographic barrier formed by the Taurus Mountains, together with the bioclimatic aridity signal of interior regions, appears to constrain the species from expanding into Central and Eastern Anatolia; while this pattern is consistent with the known hydrological requirements of the species, it should be noted that the models were calibrated on bioclimatic rather than direct hydrological variables.

##### 3.1.1.2 Scenario and Temporal Comparisons

**FIGURE 3:**
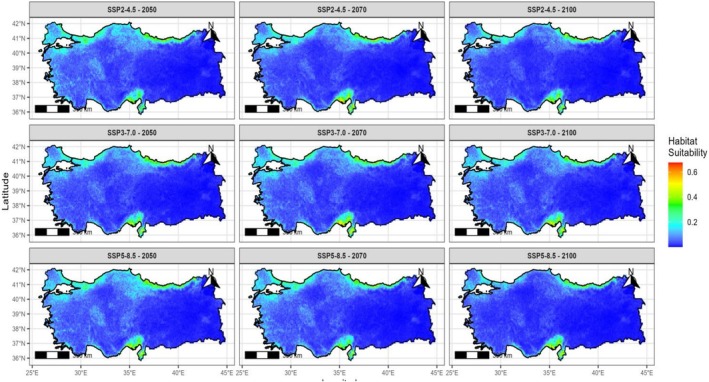
Projected future habitat suitability for *Eichhornia crassipes* in Turkey across three distinct shared socioeconomic pathways (SSP2‐4.5, SSP3‐7.0 and SSP5‐8.5) and three temporal horizons (2050, 2070 and 2100). The projections were generated utilising the high‐resolution consensus ensemble modelling framework. The colour gradient indicates potential habitat suitability, ranging from 0 (blue, climatically unsuitable) to 1.0 (red, highly suitable optimal conditions).

Building upon this baseline, the comparative time‐series maps for future projections (Figure 3) show that the potential distribution of this species in Turkey remains highly spatially stable across all scenarios and timeframes examined. Notably, the shift from the moderate‐emission SSP2‐4.5 trajectory to the most extreme SSP5‐8.5 scenario did not result in any discernible expansion of the species' potential range. Projections for 2050, 2070 and 2100 consistently show that areas of high suitability along the coastal fringe are largely retained, but no new suitable habitats appear within the interior of the country.

These findings collectively indicate that *E. crassipes* has limited additional climatic expansion potential within its existing coastal habitats in Turkey, and that its distributional pattern is consistent with hydrological limitation rather than with thermal constraint, although hydrological variables were not directly incorporated as model predictors and this interpretation should therefore be regarded as inferential rather than directly tested.

#### 3.1.2 *Solanum elaeagnifolium*

##### 3.1.2.1 Current Distribution

**FIGURE 4:**
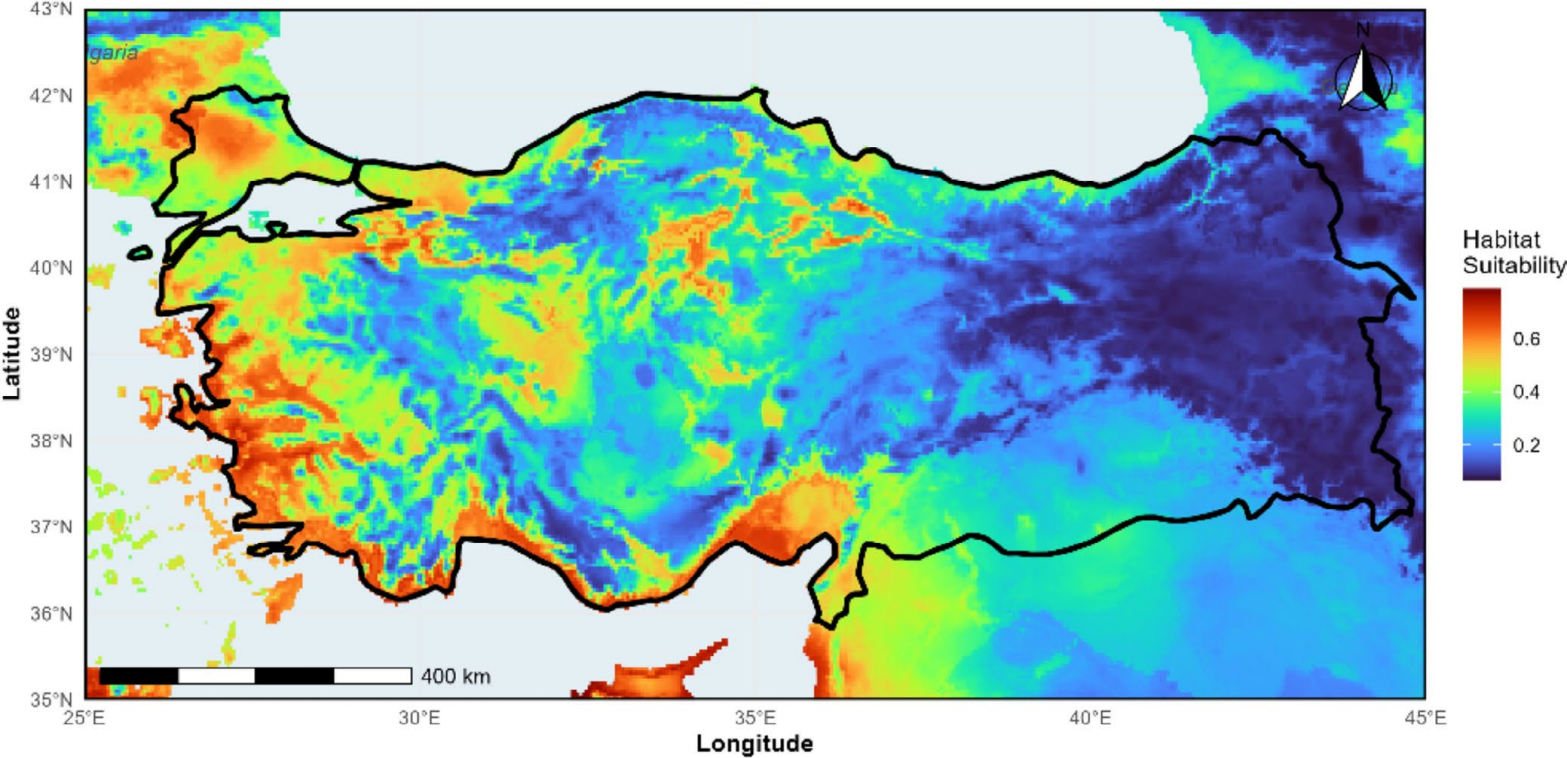
Current potential habitat suitability for *Solanum elaeagnifolium* in Turkey under baseline climatic conditions. The colour gradient from dark blue to red represents increasing habitat suitability (ranging from 0 to 1.0), with red areas indicating optimal climatic conditions for establishment.

**FIGURE 5:**
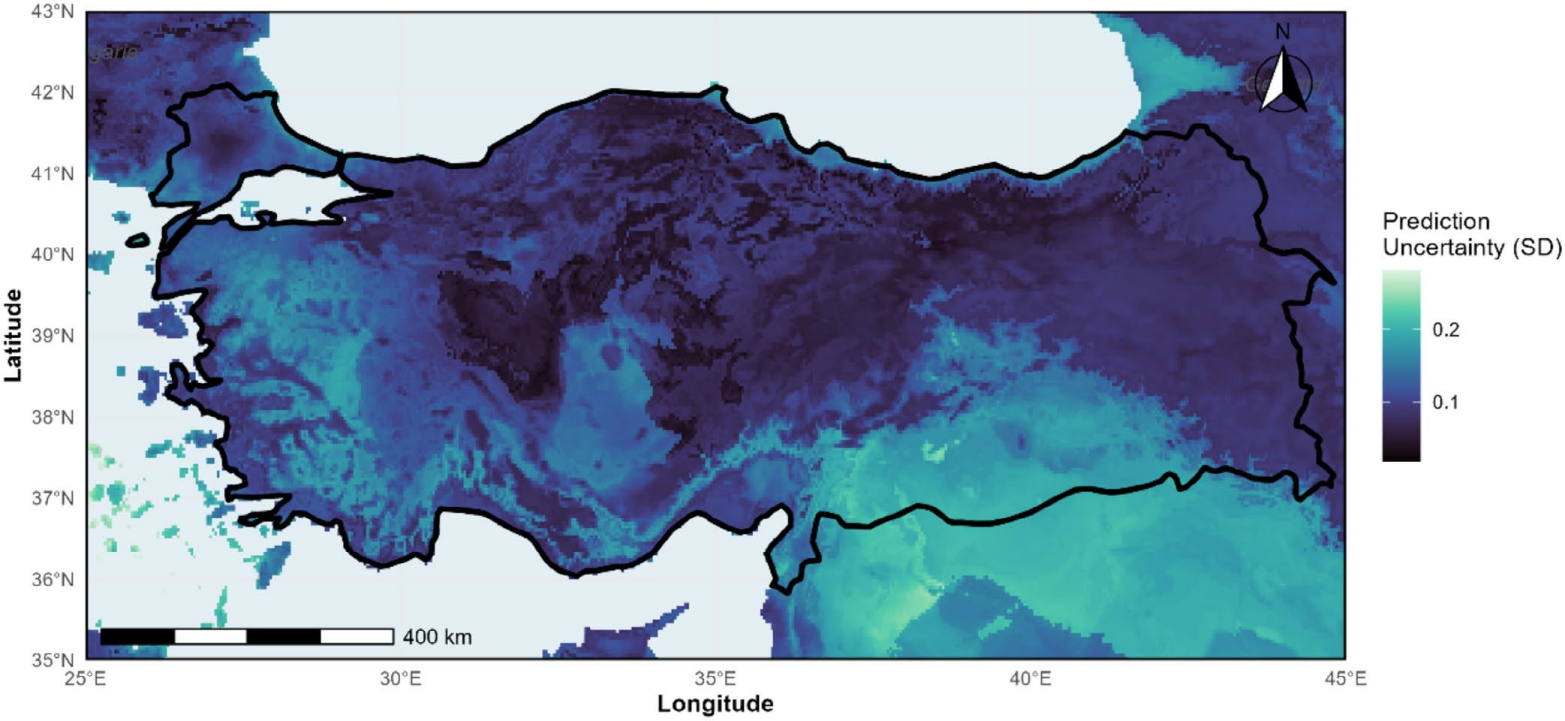
Spatial prediction uncertainty (standard deviation) associated with the ensemble modelling framework for *Solanum elaeagnifolium* . Higher standard deviation values (lighter colours) indicate geographic regions with greater variance and lower consensus among the constituent machine‐learning algorithms (MaxEnt, RF and BRT), whereas darker areas indicate high model agreement.

Initial model outputs (Figure 4) indicate that the extant potential distribution of the xerophytic *S. elaeagnifolium* is concentrated mainly along the coastal plains and transitional ecotones, with suitability across interior regions remaining comparatively restricted. The reliability of these baseline spatial patterns is corroborated by the prediction uncertainty analysis (Figure 5), which displays strong model consensus across these initial ranges.

##### 3.1.2.2 Scenario Influence and Range Dynamics

**FIGURE 6:**
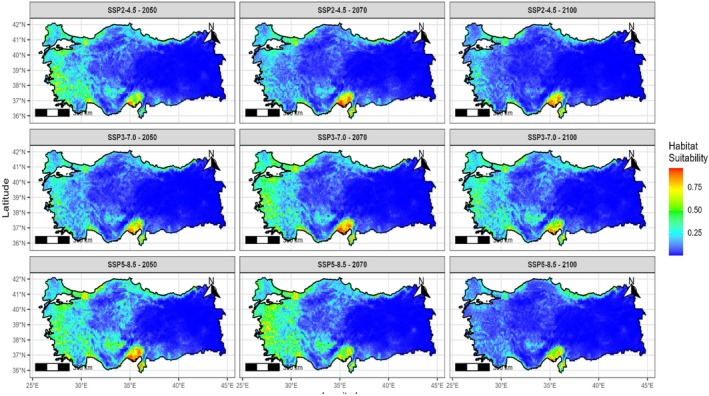
Projected future habitat suitability for *Solanum elaeagnifolium* in Turkey across three distinct shared socioeconomic pathways (SSP2‐4.5, SSP3‐7.0 and SSP5‐8.5) and three temporal horizons (2050, 2070 and 2100). The projections were generated utilising the high‐resolution consensus ensemble modelling framework. The colour gradient indicates potential habitat suitability, ranging from 0 (blue, climatically unsuitable) to 1.0 (red, highly suitable optimal conditions).

In contrast to the hydrophilic *E. crassipes* , comparative time‐series maps for future projections (Figure 6) show a dynamic and directionally explicit pattern of range expansion under the climate change scenarios examined. Under the SSP5‐8.5 trajectory, a pronounced inland shift in habitat suitability was observed, beginning from the 2050 temporal window. Areas classified as high‐suitability—depicted in darker green hues—were found to progressively retreat from coastal belts and coalesce within continental interior regions.

##### 3.1.2.3 Emergent Risk Zones (Invasion Front)

Projections for 2070 and 2100 indicate that *S. elaeagnifolium* is poised to establish a conspicuous invasion front along two principal axes of expansion:

1. The Inner Aegean Corridor: A dispersal pathway has emerged along which the species is projected to advance towards the southern areas of Uşak, Denizli and Afyonkarahisar provinces.
2. The Southeastern Anatolia (GAP) Corridor: With rising temperatures and increased aridity, the plains of Gaziantep, Kilis and Şanlıurfa are expected to move into the high habitat suitability class by 2100.

These spatial patterns clearly demonstrate that *S. elaeagnifolium* can exploit rising temperatures and intensifying drought conditions to gain a competitive advantage and that climate change is actively creating new invasion corridors for this taxon.

Examination of the current potential distribution maps for the target taxa shows that both species have extensive areas of climatic suitability across Turkey, consistent with their respective ecological strategies (Figures 1 and 4). The ‘Current’ depiction in these cartographic outputs represents not the realised, existing invasion extent of the species, but the full geographic range where climatic conditions permit establishment. The area statistics reported here—2484 km^2^ for *E. crassipes* and 37,353 km^2^ for *S. elaeagnifolium* —are based solely on the core zones within the broader suitability matrix, specifically those regions with the highest probability of establishment (suitability > 0.80). This analytical distinction between the visually depicted suitability envelope and the numerically quantified core habitat explains the apparent scaling discrepancy between the mapped representations and the tabulated area values.

**FIGURE 7:**
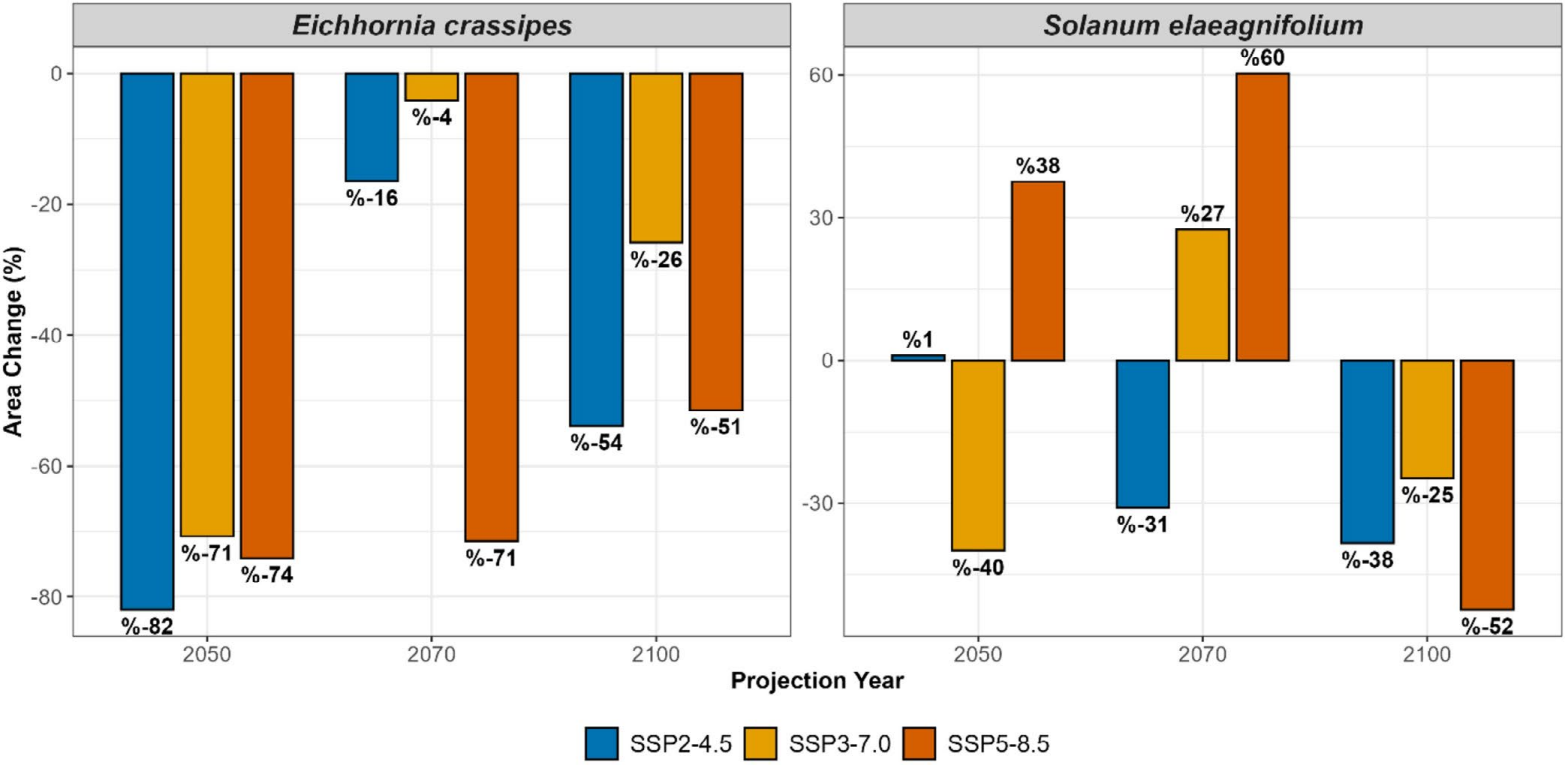
Relative percentage changes in potential habitat extent (%) for the focal taxa ( *E. crassipes* and *S. elaeagnifolium* ). The graph presents projections across three SSP scenarios (SSP2‐4.5, SSP3‐7.0 and SSP5‐8.5) and three future temporal windows.

**FIGURE 8:**
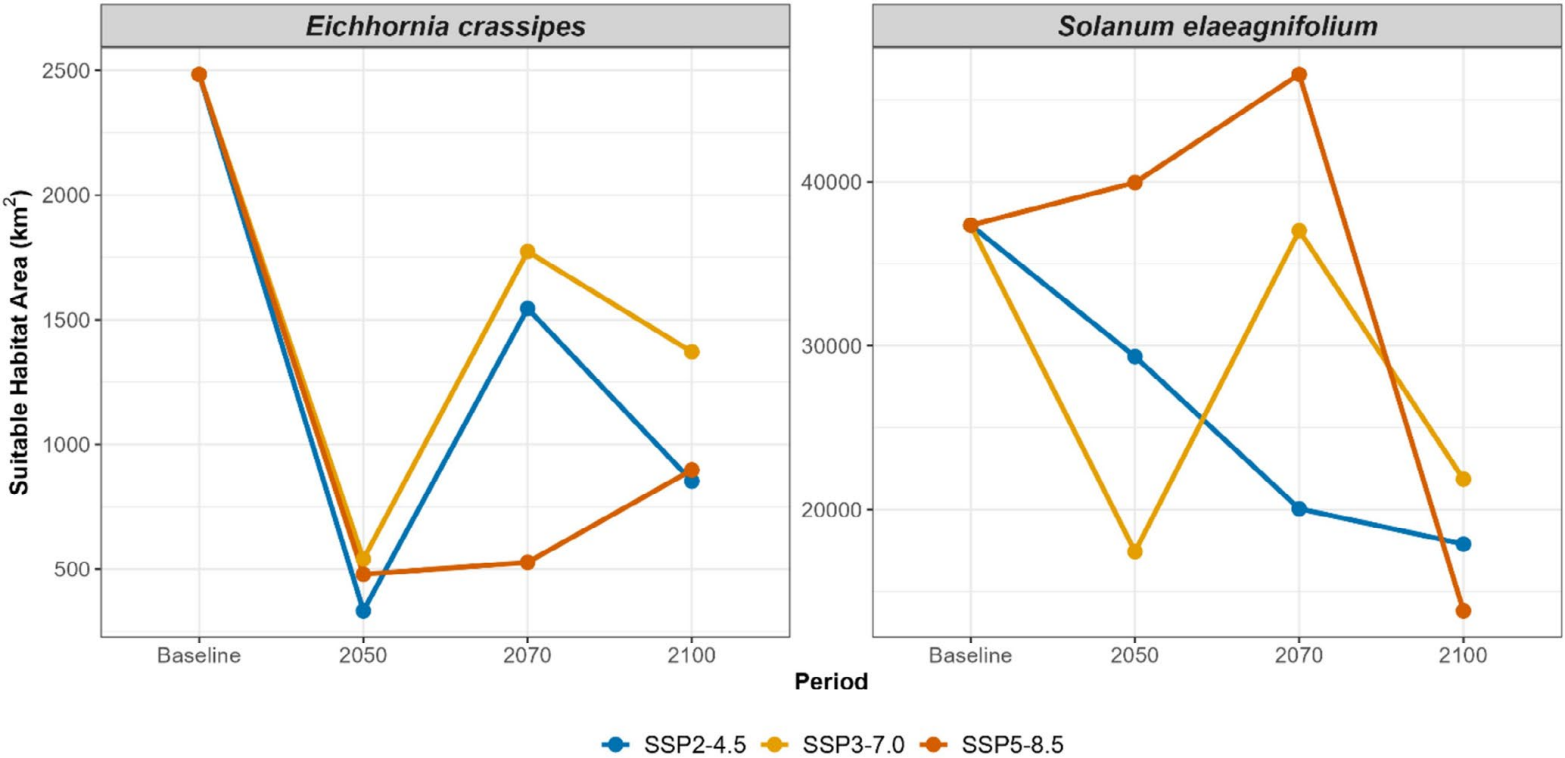
Temporal projection of suitable habitat extent (km^2^) from the baseline period to the year 2100. The trajectories illustrate the dynamic responses of the focal taxa to divergent climate forcing scenarios, including the projected thermal threshold declines for *S. elaeagnifolium* under the most extreme warming trajectories.

Temporal analysis of the projected habitat changes reveals complex, non‐linear trajectories for both focal taxa (Figures 7 and 8). For the xerophytic *S. elaeagnifolium* , the potential core habitat area initially fluctuates but experiences a pronounced expansion by 2070, peaking under the extreme SSP5‐8.5 scenario with a maximum increase of 60.3%. However, this expansion is followed by a sharp contraction towards the end of the century (2100) across all scenarios, with the core habitat declining by over 52% under the SSP5‐8.5 trajectory. In stark contrast, projections for the hydrophilic *E. crassipes* indicate a severe, immediate reduction in highly suitable core habitats by 2050, with areal contractions exceeding 70% under all emission scenarios. Although a marginal recovery is observed by 2070—most notably under the SSP3‐7.0 scenario where the loss is mitigated to −4.1%—the overall long‐term trajectory for *E. crassipes* remains decisively negative through 2100.

The figures show relative changes calculated with reference to the current climatic baseline core areas for each species (2484 km^2^ for *E. crassipes* and 37,353 km^2^ for *S. elaeagnifolium* ).

Examination of areal changes under the suite of climate scenarios reveals that *E. crassipes* faces a consistent trajectory of habitat reduction across all projections. The least severe impact is observed under the SSP3‐7.0 scenario in 2070, where the core habitat decline is buffered at −4.09%, yet the overall trend remains firmly negative, underscoring the species' vulnerability to projected environmental and hydrological shifts. In contrast, the most pronounced expansion for the xerophytic *S. elaeagnifolium* —a notable +60.31%—occurs under the extreme SSP5‐8.5 scenario in the 2070 timeframe. However, projections for 2100 indicate a contraction across all scenarios, with the core habitat decreasing by 38.38% under the moderate SSP2‐4.5 pathway. This suggests that, while initial warming creates new invasion corridors for *S. elaeagnifolium* , the extreme thermal and arid conditions projected for the end of the century may ultimately exceed even its robust physiological thresholds.

## 4 Discussion

The findings of this study provide empirical evidence that the effects of climate change on biological invasions are neither unidirectional nor uniform; instead, they vary significantly according to species‐specific physiological tolerances, ecological strategies and hydrological constraints. Previous research has shown that climate change does not automatically or universally favour the expansion of all invasive alien taxa. Bellard et al. (2013) suggested that global warming is likely to create distinct ‘winners and losers’ among invasive species, with increasing thermal extremes and intensified drought stress opening new dispersal corridors for some taxa while making existing ecological barriers more difficult to overcome for species sensitive to water limitation. Similarly, Early and Sax (2014), in their study of climatic niche dynamics, demonstrated that range expansion potential is limited not only by rising temperatures but also by the fundamental ecological requirements of the species—particularly hydrological thresholds and thermal tolerance limits. The results presented here concretise and spatially illustrate this theoretical framework within the specific geographic context of Turkey. Our study shows a marked asymmetry in the spatial responses of two taxa with contrasting ecological strategies: while the hydrophilic *E. crassipes* faces a persistent reduction in suitable habitat due to increased hydrological stress, the xerophytic *S. elaeagnifolium* exhibits a more complex, non‐linear response—experiencing a transient expansion phase before encountering thermal and arid conditions by 2100 that ultimately constrain its distributional potential. These spatial projections provide strong statistical evidence that global warming does not represent a universal ‘expansion opportunity’, and that for many taxa, the initial competitive advantages conferred by rising temperatures may be ultimately offset by the long‐term exceeding of physiological thresholds.

The model outputs indicate that, while *E. crassipes* currently maintains high habitat suitability along the Mediterranean and Aegean coastal littorals of Turkey, its future potential is increasingly threatened by climatic shifts. Contrary to the initial expectation of range expansion, our quantitative data reveal a severe contraction in suitable habitats across all tested emission scenarios (Table 4). This trend suggests that the ecological niche of *E. crassipes* in Turkey is consistent with vulnerability to hydrological and thermal stressors associated with climate change, rather than being solely limited by frost events; as the models did not directly incorporate hydrological predictors (e.g., distance to water bodies, wetland extent), this interpretation is drawn from the spatial pattern of suitability loss and from the species' known ecology rather than from direct variable‐level evidence within the model. Although niche conservatism might suggest that the species would persist where thermal constraints are reduced, our results demonstrate that the projected increases in drought severity and the degradation of wetland habitats—which are critical for this hydrophilic taxon—far outweigh any potential benefits from milder winters, leading to a substantial reduction in its invasion potential by the end of the century. Although invasive taxa often exhibit niche conservatism, our model outputs for *E. crassipes* indicate that its potential distribution within Turkey is significantly constrained by projected climatic shifts rather than climatic saturation. Although rising temperatures might theoretically enhance thermal conditions for this tropical‐origin species, our results demonstrate that the simultaneous decline in water availability and increased evaporative demand represent insurmountable barriers, restricting its presence to highly fragmented aquatic networks. These findings corroborate the global assessments by Kriticos and Brunel (2016), which emphasise that the distribution of this species is primarily limited by water availability rather than thermal regimes alone. Consequently, under projected climate change, *E. crassipes* should be viewed not as a spatially expansive invader but as a species facing severe contraction of its niche envelope. The primary ecological risk associated with this taxon in Turkey, therefore, shifts from broad‐scale geographic invasion to the intensification of ecological pressure within the remaining, highly fragmented micro‐refugia, where reduced water levels further concentrate the impacts of the species on local ecosystem functioning. In contrast, the results for *S. elaeagnifolium* indicate that climate change acts as a complex driver for range dynamics rather than a unidirectional expansion catalyst. Although the models show an initial invasion front moving from coastal areas towards the Inner Aegean and Southeastern Anatolia—peaking with a notable +60.31% expansion by 2070 under the SSP5‐8.5 scenario—this is followed by a widespread contraction across all scenarios by 2100 (Table 4). This expansion phase is consistent with the ecological strategies typical of *S. elaeagnifolium* , such as its deep, extensively branched root system and high drought tolerance. However, the subsequent decline suggests that while drought stress initially acts as a selective filter that provides a significant competitive advantage in water‐limited environments, the extreme thermal and arid conditions projected for the end of the century eventually exceed the physiological thresholds of the species. Thus, *S. elaeagnifolium* should be interpreted as a taxon that leverages transient climate‐driven opportunities, only to face significant distributional constraints once climatic stressors reach extreme levels. The progressive enhancement of habitat suitability projected for transitional zones such as Uşak, Denizli and Afyonkarahisar through 2070 aligns with the documented expansion capacity of this species within the broader Mediterranean Basin. However, one of the more distinctive and theoretically significant findings from our study concerns the phenomenon of a thermal stress threshold. The shift from a peak expansion of +60.31% by 2070 to a contraction of −52.39% by 2100 under the SSP5‐8.5 trajectory strongly suggests that even this markedly xerophytic taxon encounters its upper physiological thermal limits under the most extreme warming projections. Conversely, the more moderate contraction of −38.38% observed under the SSP2‐4.5 scenario by 2100 indicates that the implementation of aggressive emissions' mitigation measures may play a critical role in stabilising or at least buffering future invasion risk associated with this species.

**TABLE 4:** Potential area changes (km^2^ and %) for the focal invasive alien species under different CMIP6 climate scenarios.

Species	Scenario	Year	Potential area (km^2^)	Change (%)	Status
*Eichhornia crassipes*	SSP2‐4.5	2050	333.16	−81.98	Decrease
		2070	1545.15	−16.44	Decrease
		2100	852.85	−53.88	Decrease
	SSP3‐7.0	2050	540.23	−70.78	Decrease
		2070	1773.53	−4.09	Decrease
		2100	1370.87	−25.86	Decrease
	SSP5‐8.5	2050	479.29	−74.08	Decrease
		2070	527.21	−71.49	Decrease
		2100	897.33	−51.47	Decrease
*Solanum elaeagnifolium*	SSP2‐4.5	2050	29340.01	+1.01	Increase
		2070	20054.31	−30.95	Decrease
		2100	17897.53	−38.38	Decrease
	SSP3‐7.0	2050	17436.27	−39.97	Decrease
		2070	37029.33	+27.49	Increase
		2100	21854.73	−24.76	Decrease
	SSP5‐8.5	2050	39959.62	+37.58	Increase
		2070	46563.05	+60.31	Maximum increase
		2100	13829.14	−52.39	Decrease

The projected climatic aridification across Turkey is expected to create transient ecological niches, facilitating the proliferation of *S. elaeagnifolium* during the mid‐21st century. The expansion forecast for the Southeastern Anatolia Region (GAP) in particular poses a significant threat to agricultural productivity and regional food security through 2070. The allelopathic properties and aggressive competitive vigour exhibited by *S. elaeagnifolium* have the potential to substantially exacerbate yield losses in strategically vital crops, including cotton and cereals, during this peak invasion phase. Although our projections indicate a subsequent contraction of its potential range by 2100, the socio‐economic repercussions generated by this mid‐century expansion—acting through intensified biological invasion—could trigger profound and far‐reaching impacts on regional agriculture, necessitating urgent and proactive management strategies.

The most salient and operationally significant finding from this study is that managing invasive alien species amid accelerating climate change cannot rely on monolithic or static intervention frameworks. For *E. crassipes* , the projected spatial contraction—restricting its potential range primarily to fragmented micro‐refugia—indicates that management strategies should shift from broad‐scale eradication to the sustained monitoring of established populations and preservation of vulnerable aquatic habitats. In contrast, the rapid and directionally explicit range expansion forecast for *S. elaeagnifolium* through 2070 requires urgent implementation of stringent quarantine enforcement, robust early warning systems and proactive, spatially targeted interventions informed by high‐resolution regional risk mapping. Crucially, our projections also demonstrate that the mid‐century expansion phase of *S. elaeagnifolium* is transient, as climatic stressors are projected to surpass its physiological limits by 2100. This reinforces the need for dynamic management protocols that adapt to the shifting invasion risk over time. This differentiated approach aligns with contemporary risk assessment frameworks that advocate integrating climate projections into invasive species risk evaluation and prioritisation protocols (Hellmann et al. 2008; Bellard et al. 2013). Notably, our results corroborate global‐scale analyses suggesting that climatic niche shifts for many invasive plant taxa are considerably more constrained than previously assumed, with species largely persisting within the boundaries of their pre‐existing fundamental niche envelopes (Petitpierre et al. 2012).

Overall, the findings of this study demonstrate that climate change influences biological invasions not merely as a quantitative function of areal expansion or contraction, but as a species‐specific, directionally explicit process of spatial reconfiguration. By integrating CMIP6 climate projections with species‐specific physiological thresholds, this research reveals that the invasion risk is highly dynamic, characterised by transient expansion phases followed by long‐term distributional constraints. These results underscore the imperative that future invasive species risk assessments move beyond simplistic correlative approaches, integrating climate projections with a nuanced understanding of the focal taxon's inherent ecological strategies to foster more mechanistic and context‐sensitive evaluative frameworks.

Although the potential range dynamics of the focal invasive taxa examined in this study are based on robust statistical and theoretical foundations, certain methodological caveats require explicit acknowledgement. It is important to distinguish between two conceptually distinct sources of uncertainty addressed in this study. The first, algorithmic uncertainty, arises from differences among the modelling techniques themselves (MaxEnt, RF and BRT), each of which makes different statistical assumptions about the species–environment relationship; this source of uncertainty is explicitly quantified in the present study through the ensemble standard deviation maps (Figures 2 and 5), where areas of high inter‐model agreement indicate greater confidence and areas of disagreement flag regions requiring cautious interpretation. The second, climate‐model (structural) uncertainty, arises from inter‐GCM variability in how future climate itself is simulated, independent of any SDM algorithm. This second source of uncertainty is not addressed by the ensemble SDM framework and remains a distinct limitation of the present study: the reliance on a single general circulation model (GCM)—specifically, the MPI‐ESM1‐2‐HR—may have limited the full representation of variance associated with future climatic uncertainties. Nevertheless, the demonstrated fidelity of this GCM in simulating the semi‐arid climatic extremes characteristic of the Mediterranean Basin supports the reliability and regional relevance of the spatial projections obtained here. Furthermore, while the 5 arc‐minute spatial resolution effectively captures macro‐climatic shifts, it may not fully resolve the fine‐scale micro‐hydrological conditions critical for hydrophilic taxa like *E. crassipes* , necessitating finer scale regional models in future studies. Future investigations that validate these findings through multi‐GCM ensembles and seek to incorporate biotic interactions—such as competition and predation—into the modelling architecture will further refine the precision and predictive acuity of regional‐scale risk assessments. In conclusion, despite these inherent methodological constraints, the insights generated from our spatial projections offer a critical foundation for developing adaptive management strategies, ultimately contributing to a more nuanced understanding of how biological invasions will intersect with the climate‐driven reconfiguration of ecosystems in the Mediterranean Basin.

In conclusion, despite these inherent methodological constraints, the insights generated from our spatial projections offer a critical foundation for developing adaptive management strategies, ultimately contributing to a more nuanced understanding of how biological invasions will intersect with the climate‐driven reconfiguration of ecosystems in the Mediterranean Basin.

## 5 Conclusion

This study clarifies that the effects of climate change on biological invasions are neither linear nor uniform; instead, they produce complex responses contingent upon species‐specific physiological tolerances and hydrological dependencies. Comparative findings from the hydrophilic *E. crassipes* and the xerophytic *S. elaeagnifolium* provide clear empirical evidence that global warming does not automatically confer a universal expansion advantage to all invasive taxa. Our results indicate that *E. crassipes* faces severe habitat contraction, with its potential distribution increasingly restricted to fragmented micro‐refugia, where it exerts intensified pressure on vulnerable freshwater systems. In contrast, *S. elaeagnifolium* exhibits a dynamic, non‐linear range response: an initial mid‐century expansion phase towards interior agricultural landscapes, followed by a widespread contraction by 2100 as climatic stressors surpass its physiological thresholds. These contrasting dynamics highlight that biological invasions pose multidimensional risks to agricultural production, food security and rural economic vitality. Consequently, invasive species risk assessment must adopt an integrative approach that transcends simplistic areal quantification, incorporating the direction, velocity and functional characteristics of ecosystem change. For taxa with transient expansion potential like *S. elaeagnifolium* , proactive management—supported by robust early warning systems and spatially explicit risk mapping—is an urgent priority. In conclusion, this study demonstrates that climate‐driven biological invasions are inherently dynamic processes of spatial reconfiguration. This perspective underscores the need for adaptive, species‐centric management frameworks that account for both the immediate opportunities and the long‐term physiological constraints imposed by a rapidly changing climate.

## Author Contributions

**Nihat Tursun:** conceptualization (lead), data curation (lead), formal analysis (lead), investigation (equal), project administration (lead), writing – original draft (lead), writing – review and editing (equal). **İlhan Üremiş:** writing – review and editing (equal). **Soner Soylu:** methodology (supporting), writing – review and editing (equal). **Ahmet Uludağ:** writing – review and editing (equal).

## Funding

The authors have nothing to report.

## Conflicts of Interest

The authors declare no conflicts of interest.

## Supporting information


**Figure S1:** Pearson correlation matrix of the initial 19 bioclimatic variables. To prevent multicollinearity in the modelling process, highly correlated predictors (VIF > 5 and |*r*| > 0.75) were identified and subsequently removed. The colour gradient and numerical values indicate the strength and direction of the correlation coefficients. Following this elimination, the final variables retained for the models were Bio1: Annual mean temperature; Bio3: Isothermality; Bio4: Temperature seasonality; Bio8: Mean temperature of wettest quarter; Bio9: Mean temperature of driest quarter; Bio14: Precipitation of driest month; Bio15: Precipitation seasonality; Bio19: Precipitation of coldest quarter.

## Data Availability

The raw data supporting the conclusions of this manuscript are openly available in public repositories. Species occurrence data for *Eichhornia crassipes* and *Solanum elaeagnifolium* were sourced from the Global Biodiversity Information Facility (GBIF.org 2026). The baseline and future climate projection data sets (MPI‐ESM1‐2‐HR model, CMIP6) were obtained from the WorldClim 2.1 database (www.worldclim.org). The custom R scripts used for multicollinearity analysis (VIF/Pearson) and the ensemble modelling framework (MaxEnt, RF, BRT), together with the cleaned occurrence data and model validation results, are openly available on Zenodo at https://doi.org/10.5281/zenodo.21259379 (Tursun et al. 2026).
