# Supplementary material for: Comparative Distribution Projections of Hydrophilic and Xerophytic Invasive Species in Turkey Under CMIP6 Climate Scenarios
Source: Ecol Evol. 2026 Aug 15;16(8):e74172. doi: 10.1002/ece3.74172 (PMC13476759; doi:10.1002/ece3.74172)
Supplement: Supplementary file 1 [file ECE3-16-e74172-s001.docx]

**Figure S1.** Pearson correlation matrix of the initial 19 bioclimatic variables. To prevent multicollinearity in the modelling process, highly correlated predictors (VIF > 5 and |r| > 0.75) were identified and subsequently removed. The color gradient and numerical values indicate the strength and direction of the correlation coefficients. Following this elimination, the final variables retained for the models were: Bio1: Annual Mean Temperature; Bio3: Isothermality; Bio4: Temperature Seasonality; Bio8: Mean Temperature of Wettest Quarter; Bio9: Mean Temperature of Driest Quarter; Bio14: Precipitation of Driest Month; Bio15: Precipitation Seasonality; Bio19: Precipitation of Coldest Quarter.
